# Metabolomics Applied to Pediatric Asthma: What Have We Learnt in the Past 10 Years?

**DOI:** 10.3390/children10091452

**Published:** 2023-08-25

**Authors:** Valentina Agnese Ferraro, Stefania Zanconato, Silvia Carraro

**Affiliations:** Unit of Pediatric Allergy and Respiratory Medicine, Women’s and Children’s Health Department, University of Padova, 35122 Padova, Italy

**Keywords:** pediatric asthma, metabolomics, predictive medicine, asthma endotypes, pharmaco-metabolomics

## Abstract

**Background**: Asthma is the most common chronic condition in children. It is a complex non-communicable disease resulting from the interaction of genetic and environmental factors and characterized by heterogeneous underlying molecular mechanisms. Metabolomics, as with the other omic sciences, thanks to the joint use of high-throughput technologies and sophisticated multivariate statistical methods, provides an unbiased approach to study the biochemical–metabolic processes underlying asthma. The **aim** of this narrative review is the analysis of the metabolomic studies in pediatric asthma published in the past 10 years, focusing on the prediction of asthma development, endotype characterization and pharmaco-metabolomics. **Methods**: A total of 43 relevant published studies were identified searching the MEDLINE/Pubmed database, using the following terms: “asthma” AND “metabolomics”. The following filters were applied: language (English), age of study subjects (0–18 years), and publication date (last 10 years). **Results and Conclusions**: Several studies were identified within the three areas of interest described in the aim, and some of them likely have the potential to influence our clinical approach in the future. Nonetheless, further studies are needed to validate the findings and to assess the role of the proposed biomarkers as possible diagnostic or prognostic tools to be used in clinical practice.

## 1. Introduction

In the past few decades, a growing contribution to the study of non-communicable diseases has been provided by omic sciences that, thanks to the application of high-throughput technologies, enable the overall characterization of molecular profiles associated with conditions of interest [1]. Metabolomics systematically analyzes metabolites (small molecules that are the intermediate or end products of metabolic reactions) enabling the characterization of the biochemical–metabolic arrangement of a biological sample, which is affected by both genetic information and environmental influences [2]. Therefore, metabolomics has promising application in the investigation of complex diseases like asthma, the pathogenesis of which is influenced by both environmental and genetic factors [3].

Asthma is the most common chronic condition in children worldwide according to the World Health Organization (https://www.who.int/news-room/fact-sheets/detail/asthma (accessed on 20 August 2023)). Complex interactions between multiple factors underlie asthma, including genetics, the environment, infection, immunity and nutrition, with a key role played by early-life exposures [4,5]. Moreover, several pathways characterized the pathophysiology of asthma such as airway inflammation, airway hyper-responsiveness, reversible airflow obstruction, and airway remodeling [6], but the pathogenetic mechanisms have not been fully understood yet.

A great number of studies have investigated different aspects of asthma by applying the metabolomic approach that, being guided by no a priori hypotheses, has the potential to shed light on the underlying biochemical–metabolic processes. In the past two decades, the research has moved from a generic description of the metabolic arrangement associated with asthma to the characterization of the biochemical metabolic mechanisms underlying specific aspects of asthma. Now, the research gap to be filled is how metabolomic data can guide the development of simple diagnostic or prognostic tools to be use in clinical practice.

**Figure 1:**
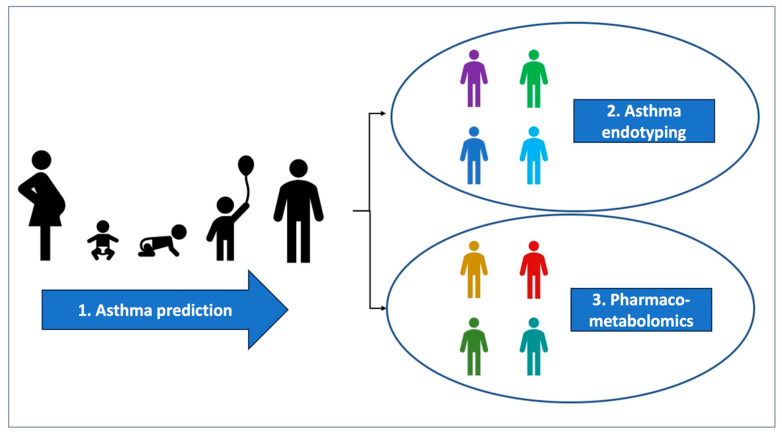
Three areas of research discussed in this review. The first area is early identification of metabolomic profiles predictive of asthma; the other two areas concern children with established asthma in whom metabolomics might help characterizing different endotypes as well as metabolic profiles predictive of therapy response or adverse effect (pharmaco-metabolomics).

The aim of the present manuscript is to narratively review the pediatric studies, published in the last 10 years, that investigate the role of metabolomics in the prediction of asthma development, in the characterization of asthma endotypes and in the analysis of drug effects and response to treatment (pharmaco-metabolomics) (Figure 1).

## 2. Materials and Methods

In this narrative review, we included relevant published studies identified searching MEDLINE/Pubmed using the following terms: “asthma” AND “metabolomics”. The following filters were applied: language (English), age of study subjects (0–18 years), publication date (last 10 years), and human studies. No filters about study design were applied.

Among the retrieved papers, we considered all those referable to one of the following three research areas: early prediction of asthma development, pediatric asthma endotyping and pharmaco-metabolomics.

Data extraction and curation were performed by V.A.F. and S.C.; only full papers were included.

## 3. Results

Our search identified 43 pediatric studies on the role of metabolomics in the prediction of asthma development, in the characterization of asthma endotypes and in the analysis of drug effects and response to treatment (pharmaco-metabolomics).

### 3.1. Metabolomics in Asthma Prediction

A promising field of clinical application for metabolomics is the prediction of asthma through the identification of early metabolic profiles (at birth or in the first years of life) associated with the following development of asthma (Table 1). 

Brustad et al. [7,8], in a recent paper, analyzed the effect of maternal diet on the risk of developing allergic diseases (including asthma) in children belonging to the COPSAC_2010_ mother–child cohort. The authors focused on the identification of specific vertically transferred metabolites identified in dried blood spot (DBS) samples collected 2–3 days after birth (n = 678 children). A principal component analysis of 10 vertically transferred metabolites was performed, and the presence of coffee-related metabolites (paraxanthine and caffeine) was associated with a decreased risk of asthma at 6 years of age [7]. The authors hypothesized, therefore, a role for early nutritional intake in long-term health, suggesting the DBS metabolomic-based approach as a promising strategy to study the metabolites’ vertical transmission from mother to child [7].

A study from our group [9] demonstrated that children who develop recurrent wheezing not preceded by acute bronchiolitis, compared to those without recurrent wheezing, had a specific urinary metabolomic signature at birth (with lower levels of xanthine, uric acid, leucine, L-tyrosine, L-pyroglutamic acid and L-ornithine). This finding suggests the presence of early biochemical–metabolic features that put children at risk of developing recurrent wheezing. On the other hand, no association was found between the metabolomic profile at birth and the development of both acute bronchiolitis and post-bronchiolitis recurrent wheezing. 

Chawes et al. [10] demonstrated in two separate birth cohorts (COPSAC2000 and COPSAC2010) that the urinary metabolomic profile in healthy infants 4 weeks old born from mothers with asthma showed some metabolic features associated with the development of asthma in the first 6 years of life: (a) increased taurochenodeoxycholate-3-sulfate (a bile acid compound emerging as regulator of the gut microbiome); (b) increased 3-hydroxytetradecanedioic acid (fatty acid), which probably reflects a perturbed pro-inflammatory fatty acid metabolism; (c) reduced levels of a glucoronidated steroid compound, which probably is associated with an abnormal stress response and systemic inflammation. In the same cohorts, the authors analyzed also blood collected a few days after birth (as dried blood spots) to study if a specific metabolomic arrangement characterized those born by cesarean section [21]. In these children, compared to those born via vaginal birth, a metabolic fingerprint characterized by lower levels of tryptophan catabolites and bile acids was reported. The metabolic arrangement associated with cesarean section correlated with the diagnosis of asthma at 6 and 7 years of age, and the authors hypothesized that such a metabolic profile could be the result of the altered gut microbiota that characterizes neonates born by cesarean section. The authors suggest that the reported metabolomic perturbances could have a role in the pathogenetic relationship between cesarean section and asthma development.

In children 6 months old belonging to cohorts COPSAC2010 and VDAART, applying a lipidomic approach, the authors analyzed sphingolipids because of their potential role in airway hyperresponsiveness, inflammation and remodeling [11]. The authors found that reduced levels of ceramides and sphingomyelin were associated with early onset (before the age of 3 years) of wheezing but not with the diagnosis of asthma at 6 years of age [11]. Moreover, Rago et al. [22] showed in the COPSAC2010 cohort that maternal fish oil supplementation affected child metabolome, and this fish oil-related metabolic profile at age 6 months was significantly associated with a reduced risk of asthma by age 5. This metabolic profile was characterized by lower levels of the n-6 LCPUFA (longchain polyunsaturated fatty acids) pathway-related metabolites and saturated and monounsaturated long-chain fatty acids-containing compounds, lower levels of metabolites of the tryptophan pathway, and higher levels of metabolites in the tyrosine and glutamic acid pathway. 

Moving beyond the first year of life, Chiu et al. [12] performed a longitudinal analysis of nuclear magnetic resonance (NMR) spectroscopy-based metabolomic profiles of urine samples collected in children 1 to 4 years of age, finding an association between dimethylamine overtime levels and the diagnosis of asthma at the age of 4. Given that dimethylamine is a biogenic amine mainly formed from trimethylamine N-oxide (TMAO), a compound produced by bacteria in the intestine, the authors speculate that this metabolite may be indicative of the role of environmental changes and microbial metabolism in asthma development [12]. 

In addition, in children 2 to 5 years old with recurrent wheezing (>3 wheezing episodes in the previous year), the metabolomic analysis of urine samples enabled the discrimination of children bound to develop asthma (early onset asthma) from those affected by transient wheezing, as the former is characterized by metabolites including products of tryptophan and fatty acid metabolism [13].

Taken together, all these studies suggest that metabolomic perturbances detectable in early stages of life, even before the first appearance of respiratory symptoms, might predict the following development of asthma and guide the early identification of high-risk subjects for whom targeted preventive strategies could be developed.

A number of studies applied metabolomics also to investigate the relationship between acute bronchiolitis and the following development of recurrent/persistent wheezing and asthma. 

Barlotta et al. [14] found that the urinary metabolomic profile in infants with acute viral bronchiolitis can discriminate children who develop recurrent wheezing in the following two years, with a central role in such discrimination for metabolites involved in citric acid cycle (isocitrate, citric acid, and oxoglutaric acid). How this cycle may influence the pathophysiology of respiratory tract diseases is not clear, but its alteration may be a sign of disrupted cellular energy metabolism, reflecting a condition of stress and increased anaerobic glycolysis [14]. 

More recently, a significant contribution to this research field has been provided by the 35th Multicenter Airway Research Collaboration (MARC-35) study that included 921 infants younger than 1 year hospitalized in 17 centers across the USA for acute bronchiolitis during three epidemic seasons from 2011 to 2014. At recruitment, nasopharyngeal airway specimens were collected and later analyzed through omic approaches. Recruited children were followed up to the age of 6 to identify those who developed recurrent wheezing and asthma. The metabolomic analysis of nasopharyngeal samples allowed the characterization of a metabotype with high levels of amino acids and low levels of polyunsaturated fatty acids (PUFA), which were associated with a significant risk of developing recurrent wheezing and asthma [15]. In addition, nasopharyngeal samples were analyzed through a lipidomic approach, and a cluster analysis of the obtained spectra enabled the characterization of four clusters [16]. One of these clusters (including children with history of breathing problems, eczema, parental history of asthma and eczema, high proportion of IgE sensitization and of rhinovirus infection) was associated with a significant higher risk of recurrent wheezing and asthma (at 3 and 6 years of age, respectively). From a lipidomic standpoint, these children, compared to those belonging to the other clusters, were characterized by a lower abundance of sphingolipids and PUFA and higher abundance of lysophosphatidylcholine.

Raita et al. [17], within the subset of infants with Respiratory Syncytial Virus (RSV) bronchiolitis, could discriminate four clusters considering clinical variables together with data derived from the metabolomic and microbiomic analysis of nasopharyngeal samples. More in detail, the cluster characterized by a high proportion of parental asthma, IgE sensitization, coinfection with rhinovirus, higher abundance of S. pneumoniae and M. catarrhalis, and higher IFN-α and -γ response was significantly associated with the risk of recurrent wheezing resulting in asthma. 

Likewise, within the subset of infants with rhinovirus (RV) infection, four clusters were identified through the integrated analysis of clinical data and data derived from analysis of cytokine profile, microbiome profile and metabolomic profile, and one of these clusters was associated with a significantly higher risk of recurrent wheezing and asthma [19]. As for metabolomics, the role of lipid metabolites (e.g., sphingolipids and arachidonate) was predominant in the between-cluster discrimination. 

Moreover, in a subset of the whole cohort, the authors applied a dual transcriptomic approach (metagenomics and transcriptomics) together with a metabolomic approach, identifying an interplay among microbial profile and host response which likely contributes to asthma development [20]. 

Finally, in 744 infants who underwent both genotyping and nasopharyngeal metabolome profiling, the authors [18] applied an integrated genetics–metabolomics analysis showing that genetically driven metabolites (e.g., docosapentaenoate (DPA), 1,2-dioleoyl-sn-glycero-3-phosphoglycerol, sphingomyelin) are associated with asthma development and that there are genetic loci (e.g., ADORA1, MUC16) associated with both these metabolites and asthma susceptibility. These findings support the role of integrated genetics-metabolomics analysis as an advanced research tool to investigate the functional mechanisms of the infant bronchiolitis–childhood asthma link.

Taken together, all these studies suggest that among infants with acute bronchiolitis, in the near future, it will be possible to identify those more likely to develop recurrent wheeze and asthma on the basis of their clinical characteristics considered together with a limited number of metabolic and microbial biomarkers [23].

### 3.2. Metabolomics in Asthma Endotyping

It is nowadays widely acknowledged that asthma, although presenting with a limited number of symptoms and signs, is heterogeneous from a pathogenetic standpoint so that different endotypes can be described according to the underlying molecular mechanisms. The most commonly used endotype classifications are based on the dominant cellular type (eosinophilic, neutrophilic, paucigranulocitic or mixed profile) or on the presence of Th2 cytokines (T2-high and T2-low endotype) [24].

In addition to these classifications, which are based on the measurement of a limited number of biomarkers, a significant contribution to asthma endotyping can be provided by studies applying omic approaches. Metabolomics, in particular, can provide unbiased evidence of the ongoing biochemical–metabolic processes, enabling a better understanding of existing endotypes as well as the definition of new ones through the characterization of the underlying molecular mechanisms and the identification of biomarkers [25,26].

Sinha et al. analyzed through an NMR-based metabolomic approach the exhaled breath condensate (EBC) collected in children with asthma included in a prospective cohort at the All India Institute of Medical Sciences. The cluster analysis of the NMR spectra enabled the identification of three clusters, each characterized by a different underlying metabolic profile and associated with different phenotypic characteristics (i.e., peripheral eosinophils and neutrophils, asthma exacerbation rate, and family history of asthma) [27].

In addition, some studies applied a metabolomic approach in children with asthma to identify the metabolic arrangement associated with specific clinical features such as symptom pattern, disease severity, allergen sensitization, obesity and lung function.

Two studies investigated the relationship between the endotype and the pattern of symptoms, analyzing the frequency of exacerbations and wheezing. Cottrill et al. [28] applied a mass spectrometry (MS)-based metabolomic approach to search for a metabolomic profile (metabotype) associated with the clinical phenotype characterized by frequent exacerbations (i.e., three or more exacerbations per year). The authors found that children with exacerbation-prone asthma could be discriminated on the base of their blood metabolomic profile, which showed perturbations in the pathways of arginine, lysine and methionine. Given that acute exacerbations have a significant weight in the overall burden of childhood asthma, understanding the endotype associated with frequent exacerbations might be important with a view to developing new therapeutic strategies [28].

In addition, a study conducted in preschool children with a doctor-based diagnosis of asthma provides evidence that the gut microbiome and metabolome may have an impact on wheeze frequency in childhood asthma. The authors demonstrated an association between the fecal metabolic profile, which was enriched in histidine metabolites and relatively independent of the microbiome, and the frequency of wheezing [29].

Taken together, all these studies suggest the possible role of metabolomics in the identification of asthmatic children with frequent exacerbations. 

Regarding the association between endotype and asthma severity, Fitzpatrick et al. [30] analyzed, through an NMR-based metabolomic approach, blood samples collected in a well-characterized group of asthmatic children with severe steroid insensitive asthma (demonstrated by the lack of a significant clinical response to intramuscular triamcinolone). The authors found that these children could be discriminated from children with non-severe asthma on the basis of their metabolomic profile, which was enriched in metabolites and indicative of increased oxidative stress. The authors further looked into the mechanisms related with corticosteroid resistance, finding that children with severe steroid-resistant asthma were characterized by reduced synthesis and increased degradation of the antioxidant glutathione [31]. These studies suggest that the endotype of children with severe corticosteroid insensitive asthma is characterized by an imbalance between oxidative agents and anti-oxidant defenses.

Moreover, to study children with severe and non-severe asthma, an MS-based metabolomic approach was applied to EBC, a biologic fluid, non-invasively collected by cooling exhaled air, that mirrors the perturbances of the respiratory system better than systemic biological samples such as urine or blood. Children with severe asthma were characterized by a metabolic fingerprint within which emerged a relative abundance of adenosine and retinoic acid and a lack of a vitamin D metabolite [32].

A number of international multicenter studies, such as the Unbiased Biomarkers in Prediction of Respiratory Disease Outcome (U-BIOPRED) [33], the Severe Asthma Program (SARP) [34], and The Systems Pharmacology Approach to Uncontrolled Pediatric Asthma (SysParmaPediA) [35] are ongoing with the objective of further characterizing endotypes underlying severe asthma by integrating the results derived from multiple omic approaches applied to different biological samples (urine, saliva, plasma/serum, EBC, etc.). 

The integration of information coming from multi-omics platforms has been proposed also to investigate the endotypes associated with other clinical features, such as allergic sensitization and obesity [35]. 

Chiu et al. [36] demonstrated that the combined analysis of airway microbiota and serum metabolomic profile performed better than each omic platform alone in the discrimination of asthmatic children sensitized to dust mites. The same research group found that the metabolic profile in highly sensitized asthma was enriched in metabolites related to pyruvate and acetyl-CoA metabolism, with a key role played by the increased levels of acetic acid which was in turn strongly correlated with airway microbiota. Based on their data, the authors suggest that an integrated microbiome and metabolome analysis can help understand the role of host–microbial interactions in IgE-mediated childhood asthma [37]. Recently, Turi et al. [38] applied a multi-omics analysis (untargeted metabolomics, targeted analysis of oxylipins, and transcriptomics of lung tissue) to understand the molecular processes underlying allergic asthma etiology in house dust mite (HDM)-induced allergic mice. In these mice, this integrated analysis showed a downregulation of glycerophosphatidylcholines, glycerophosphatidylethanolamines, phosphosphingolipids, and glycerophosphoinositols, whereas there was an upregulation of several diacylglicerols, phosphatidylserines and glycerophosphate, providing additional insight into novel links between metabolic, immune, and neuronal signaling pathways triggered by HDM sensitization.

It is well known that obesity is a risk factor for childhood asthma development and for worse asthma control [39], but nowadays, obesity-related childhood asthma has been suggested as a distinct phenotype that is likely the expression of specific underlying airway inflammatory pathways resulting from complex gene–environment interactions [40]. In the last decade, several studies have identified distinct metabolic signatures more prevalent in obese children with asthma or in obese children with reduced asthma control [15,30]. Makrinioti et al. published a review on the application of metabolomics in subtyping obesity-related childhood asthma, concluding that the metabolic pathways of both fatty acid metabolism and glucose regulation may explain a large part of obesity-related asthma pathophysiology in children [41]. More in detail, obese children with asthma showed increased insulin resistance and lower plasma levels of histidine and glutamine, and in the subgroup of obese children with worse asthma control, lower levels of serum long-chain n3 polyunsaturated fatty acid were reported [42,43]. Moreover, increased blood adipokine and lipid levels were associated with reduced lung function [44,45]. Recently, Gomez-Llorente et al. [46] applied a multi-omics approach in the attempt to describe the endotype underlying pediatric allergic asthma associated with obesity. 

Regarding the association between endotype and lung function, a recent interesting study has been conducted in a large population of asthmatic children [47]. The metabolomic analysis applied to blood samples collected in 1151 subjects belonging to the Genetics of Asthma in Costa Rica Study (GACRS) enabled the characterization of five metabo-endotypes, which were then validated in 911 subjects belonging to the Childhood Asthma Management Program (CAMP). The described asthma metabo-endotypes differed for relevant lung function and clinical features. Among the metabolites involved in group discrimination, trygliceredes and phosholipids played a major role, supporting the involvement of a perturbed lipid metabolism in asthma pathogenesis [48,49]. The identified metabo-endotypes can therefore provide information on underlying mechanisms and pave the way to more personalized approaches to asthma management. 

### 3.3. Pharmaco-Metabolomics

Metabolomics has potential for the identification of metabolic biomarkers associated with therapy response and therefore is potentially useful to identify the subjects more likely to benefit from a specific treatment. This application of metabolomics is usually named pharmaco-metabolomics. 

So far, only a limited number of papers have applied a pharmaco-metabolomic approach in pediatric asthma [50], as shown in Table 2. 

Kelly et al. [51] demonstrated in a cohort of asthmatic children (CAMP, Childhood Asthma Management Program) that when evaluated longitudinally, some blood metabolites are directly (2-hydroxyglutarate) associated and some are inversely (cholesterol esters, GABA and robothymidine) associated with the decline in bronchodilator response evaluated at three timepoints between the age of 5 and 25 years (mean age 8.8, 12.8 and 16.8 years, respectively). Assuming a p-value threshold of 0.1, these findings were replicated in the independent cohort GARCS (Genetics of Asthma in Costa Rica Study). Nonetheless, within the age range 5–13 years, the authors found that in both cohorts, the reported associations were no longer significant after multiple adjustments. 

Recently, a multicenter Italian study [52] demonstrated, in children 6–17 years old with severe asthma, an association between the baseline urinary metabolomic profile and the clinical response to the anti-IgE monoclonal antibodies omalizumab, paving the way for the possible prediction of the patients more likely to benefit from this treatment. In fact, urine samples collected before starting omalizumab were analyzed through a MS-based metabolomic approach, finding that a metabolic profile enriched in dipeptides and amino acids was associated with a good response to the treatment [52]. 

In addition, pharmaco-metabolomics can be applied to evaluate the metabolic therapeutic and side effects of asthma treatment. In a small study from our group [53], a 3-week course of inhaled corticosteroids (ICS) was associated with no changes in EBC metabolic arrangement and in urinary steroid profile, which was likely because of the short treatment period. On the other hand, a recent study [54] that analyzed blood metabolomic data collected in multiple independent cohorts demonstrated a clear association between the treatment with ICS and a reduction in endogenous steroids with a significant decrease in cortisol levels demonstrated even at low ICS dose. Although the discovery and replication cohorts included only adult subjects, the findings have been validated in the CAMP cohort analyzing samples collected when participants were adolescents. This study supports the potential of metabolomics to understand the impact of a treatment, pointing to the possible contribution of pharmaco-metabolomics from the perspective of a precision medicine approach.

## 4. Limitations

The main limit of our narrative review is the lack of the rigorous approach required for systematic reviews with the inclusion of all the papers identified using the search strategy described in the method section. Moreover, analyzing the papers included, in most cases, the results have not been externally validated, thus providing good models to describe the collected data but not generalizable results. Then, a further limit is the heterogeneity of the metabolites identified, which prevents the aggregation of data and firm conclusions.

## 5. Conclusions

In conclusion, a growing number of studies applying metabolomics in pediatric asthma have been published in the past decade.

Several studies have proposed possible metabolic biomarkers that could guide the identification during the early life of children likely bound to become asthmatic patients. Within this area of research, metabolomics is also contributing to the identification of features capable of discriminating among infants with acute bronchiolitis those who will develop recurrent wheezing and asthma.

In addition, metabolomics is contributing to the characterization of the different endotypes which underlie functional and clinical features in asthmatic children.

Finally, pharmaco-metabolomics can provide instruments for the identification of children more likely to benefit from a specific treatment as well as for the characterization of the metabolic side effects of treatment, laying the foundation for more personalized therapeutic strategies.

The studies discussed in this narrative review are promising, and some have the potential to influence our daily clinical practice in the future. Nonetheless, further studies are therefore needed to externally validate the findings, confirming them in independent populations of children. Moreover, target studies are needed to measure the selected relevant molecules identified through the untargeted metabolomic approach and to assess their potential as diagnostic or prognostic tools in clinical practice.

## Figures and Tables

**Table 1:** Characteristics and main findings of the studies applying a metabolomic approach in the prediction of asthma development in children. LC-MS = liquid chromatography–mass spectrometry; NMR = nuclear magnetic resonance spectroscopy; DBS = dried blood spot.

Study	Study Design and Number of Subjects Included	Main Result	Biofluid Analyzed	Analytical Platform	Main Metabolites Involved	Putative Biochemical Pathways
Brustad et al. [7,8]	Cohort study, 678 children (newborns followed up until age 6 years)	10 diet-related metabolites in DBS samples collected 2–3 days after birth are associated with later risk of asthma	DBS	LC-MS	increased levels of coffee-related metabolites (N,N,N-trimethyl-5-aminovalerate, tryptophan betaine, stachydrine, paraxanthine, ergothioneine, homostachydrine, homoarginine, paraxanthine, caffeine, and 3-carboxy-4-methyl-5-propyl-2-furanpropanoa) were associated with a decreased risk of asthma at age 6 years	caffein-related metabolism
Carraro et al. [9]	Prospective longitudinal study, 205 children (newborns followed up for 3 years)	Urinary metabolomic profile at birth discriminate children with and without recurrent wheezing	Urine	LC-MS	lower levels of xanthine, uric acid, leucine, L-tyrosine, L-pyroglutamic acid and L-ornithine in children who develop recurrent wheezing	purine metabolism, amino acid and phenylalanine metabolism, protein metabolism, urea and nitrogen metabolism
Chawes et al. [10]	Cohort study, 411 children (age 4 weeks)	Urinary metabolomic profile discriminates 4 weeks old infants who will develop asthma	Urine	LC-MS	increased taurochenodeoxycholate-3-sulfate and 3-hydroxytetradecanedioic acid, and reduced levels of a glucoronidated steroid compound in infants who develop asthma	steroid, fatty acid metabolism and bile acids metabolism
Rago et al. [11]	Cohort study, more than 500 children (from 6 months to 6 years)	Metabolic characterization at 6 months of age of children who develop early onset asthma	Plasma	LC-MS	in early-onset asthma reduced levels of ceramides and sphingomyelins containing long-chain saturated and monounsaturated fatty acids	lipid metabolism
Chiu et al. [12]	Cross-sectional case–control study, 53 children (aged 3 to 4 years)	Longitudinal association between metabolic profile and asthma development	Urine	NMR	lower levels in asthmatic children: dimethylamine, allantoin, guanidoacetic acid, 1-methylnicotinamide	purine and amino acid metabolism, nicotinamide/nicotinate metabolism, methane metabolism and gut microbiota imbalance
Carraro et al. [13]	Prospective longitudinal study, 34 children (aged 4.0 ± 1.1 years, followed up for 3 years)	Discrimination of early onset asthma from transient wheezing in 2 to 5 years old children	Urine	LC-MS	higher levels in early-onset asthma vs. transient wheezers: 4-(4-deoxy-α-D-gluc-4-enuronosyl)-D-galacturonate, glutaric acid, 4-hydroxy nonenal, phosphatidylglycerol, 3-methyluridine, steroid O-sulfate, 5-hydroxy-L-tryptophan, 3-indoleacetic-acid, tiglylglycine, indole, cytosine, N-acetylputrescine, indole-3-acetamide, 6-methyladenine, 5-methylcytosine, N-acryloylglycine, hydroxyphenyllactic acid	tryptophan metabolism, fatty acid metabolism
Barlotta et al. [14]	Prospective longitudinal study, 52 children (less than 1 year of age, followed up for 2 years)	Identification of children who develop recurrent wheezing among those with acute viral bronchiolitis	Urine	LC-MS	isocitrate, citric acid, oxoglutaric acid, lysine, cysteine and methionine, isobutyrylglycine, N-butyrylglycine	citric acid cycle, fatty acid and amino acid metabolism, gut microbial dysbiosis
Zhu et al. [15] Fujiogi et al. [16] Raita et al. [17] Ooka et al. [18] Raita et al. [19] Zhu et al. [20] 8/23/2023 11:08:00 AM	Cohort study [15,16,17,18,19,20] 918 infants (median age, 3 months) [15,16]; 221 infants (less than 1 year) [17]; 774 infants (less than 1 year) [18]; 122 infants (less than 1 year) [19]; 244 infants (median age, 3 months) [20]	Prediction of asthma development in infants hospitalized for bronchiolitis	Naso-pharyngeal samples	LC-MS	increased amino acids and reduced level of polyunsaturated fatty acids	amino acid metabolism, fatty acid metabolism

**Table 2:** Characteristics and main findings of the studies applying pharmaco-metabolomics in pediatric asthma. LC-MS = liquid chromatography–mass spectrometry; EBC = exhaled breath condensate.

Study	Study Design and Number of Subjects Included	Main Result	Biofluid Analyzed	Analytical Platform	Main Metabolites Involved	Putative Biochemical Pathways
Kelly et al. [51]	multicenter, randomized, double-masked, clinical trial, 565 children (8.8 years followed up for 16 years)	Specific metabolites may modify the estimated effect of age on bronchodilator response	Blood	LC-MS	Metabolites directly associated with the decline in bronchodilator response: 2-hydroxyglutarate; metabolites inversely associated with the decline in bronchodilator response: cholesterol esters, GABA and robothymidine	Lipid metabolism, amino acid metabolism
Carraro et al. [52]	multicenter prospective study, 52 children (mean age 12 years)	Children with severe asthma responding to omalizumab showed a different metabolomic urinary profile at baseline compared to non-responders	Urine	LC-MS	Biomarkers increased in responders were dipeptides and amino acids (the better characterized: histamine precursor L-Histidine); biomarker increased in non-responders is uric acid	Histamine pathway, protein metabolism, urea and nitrogen metabolism
Ferraro et al. [53]	case-control design, 26 asthmatic children (median age 9.1 years) and 16 healthy children (median age 10.2)	Asthmatic children treated with a three-week course of inhaled beclomethasone dipropionate showed no significant differences in the EBC metabolic arrangement and urinary steroid profile	EBC and urine	LC-MS	After beclomethasone dipropionate treatment, there were no changes in EBC metabolomic profile nor in urinary endogenous steroid profile	None
Kachroo et al. [54]	cohort study, 14,000 individuals in four cohorts (adults and adolescents)	The largest reduction in steroid levels was associated with inhaled corticosteroid (ICS) use	Blood	LC-MS	34 corticosteroid, androgenic and pregnenolone steroid metabolites	Steroid metabolism

## Data Availability

Not applicable.
